# Predictors of mortality rate among adult HIV-positive patients on antiretroviral therapy in Metema Hospital, Northwest Ethiopia: a retrospective follow-up study

**DOI:** 10.1186/s12981-021-00353-z

**Published:** 2021-05-05

**Authors:** Kefale Lejadiss Workie, Tilahun Yemanu Birhan, Dessie Abebaw Angaw

**Affiliations:** Department of Epidemiology and Biostatistics, Institute of Public Health, College of Medicine and Health Sciences, University of Gondar, Gondar, Ethiopia

**Keywords:** Adults, AIDS, ART, Death, Ethiopia, HIV

## Abstract

**Background:**

Globally Human Immunodeficiency Virus/Acquired Immune Deficiency Syndrome (HIV/AIDS) is an ongoing public health issue associated with high morbidity and mortality. Efforts have been made to reduce HIV/AIDS-related morbidity and mortality by delivering antiretroviral therapy. However, the incidence and predictors of mortality in border areas like Metema were not investigated. This study aimed to assess predictors of mortality rate among adult HIV-positive patients on antiretroviral therapy at Metema Hospital.

**Methods:**

Retrospective follow-up study was employed among ART patients from January 1, 2013, to December 30, 2018. Data were entered in Epi-data 3.1 and exported to STATA 14 for analysis. Kaplan–Meier and Log-Rank test was used to compare survival differences among categories of different variables. In bi-variable analysis p-values < 0.20 were entered into a multivariable analysis. Multivariate Weibull model was used to measure the risk of death and identify the significant predictors of death. Variables that were statistically significant at p-value < 0.05 were concluded as predictors of mortality.

**Result:**

A total of 542 study participants were included. The overall incidence rate was 6.7 (95% CI: 5.4–8.4) deaths per 100 person-years of observation. Being male (HR = 2.4; 95% CI: 1.24–4.62), STAGE IV (HR = 5.64; 95% CI: 2.53–12.56), stage III (HR = 3.31; 95% CI: 1.35–8.10), TB-coinfection (HR = 3.71; 95% CI: 1.59–8.64), low hemoglobin (HR = 4.14; 95% CI: 2.18–7.86), BMI ≤ 15.4 kg/m^2^ (HR = 2.45; 95% CI: 1.17–5.10) and viral load > 1000 copy/ml (HR = 6.70; 95% CI: 3.4–13.22) were found to be a significant predictor for mortality among HIV patients on ART treatment.

**Conclusion:**

The incidence of death was high. Being male, viral load, those with advanced STAGE (III & IV), TB co-infected, low BMI, and low hemoglobin were at a higher risk of mortality. Special attention should be given to male patients and high public interventions needed among HIV patients on ART to reduce the mortality rate.

**Supplementary Information:**

The online version contains supplementary material available at 10.1186/s12981-021-00353-z.

## Background

Globally Human Immunodeficiency Virus/Acquired Immune Deficiency Syndrome (HIV/AIDS) is an ongoing public health issue associated with high morbidity and mortality [1, 2]. An estimated 37 million people were living with HIV across the globe; among those, 70% reside in Sub-Saharan Africa [2–4].

Accessing antiretroviral therapy (ART) has been increased since 2005, globally an estimated 21.7 million people were accessing antiretroviral therapy (ART) and 940,000 people died from AIDS-related illnesses at the end of 2017 [3, 5, 6]. Even though the introduction of Highly active antiretroviral therapy (HAART) brought a significant reduction of mortality and morbidity among patients living with HIV (PLHIV), several patients die after starting ART [7–9]. Since the success of ART depends on regular patient follow up, treatment adherence and awareness of patients regarding the relevance of ART in his life [10, 11]. AIDS-related mortality among ART experienced patients’ ranges from 4.5 to 17% [12–14]. Ethiopia is one HIV high burden Sub-Saharan country where an estimated 610,000 HIV infected people and 15,000 AIDS-related mortality occurred at the end of 2017 [15, 16]. The prevalence of HIV in Ethiopia ranges from 1.13 to 1.4% in the Amhara region [17], Metema Hospital accounts for 6.3% of HIV prevalence [10]. The survival and epidemiology of HIV in Ethiopia vary along the study periods, geographic regions, and the study population [15, 18]. Many studies in Ethiopia indicated that the incidence of mortality among HIV-infected patients who are on ART ranges from 1.75 deaths per 100 person-years of observation to 7 death per 100-person year of observation which was lowest in Jinka and highest in Asela [19–27]. Despite the risk factors of mortality among people who are on ART were researched, some settings which provide HIV/AIDS care and treatment services for a high number of seasonal daily migrant workers (Mobile workers) and movable populations like Metema Hospital were not fully addressed. There was a high magnitude of HIV in Metema Hospital compared to regional and national reports. Even though governmental and many non-governmental organizations exerted great efforts on HIV care and treatment in Metema Hospitals in all settings. However, HIV/AIDS-related mortality continues a major public health concern in the area. As a result, there is a need for local data to provide evidence for organizations working on HIV/AIDS and ART at national, regional, and district levels on risk factors of mortality among HIV/AIDS patients who are on ART. Hence, this study aimed to determine the incidence and predictors of mortality among adult HIV/AIDS patients who were on ART at Metema Hospital, Ethiopia.

## Methods

### Study setting, design, and period

Institutional based retrospective follow-up study was conducted from January 1, 2013, to December 30, 2018, in Metema Hospital, which is located in west Gondar province of the Amhara National Regional State. It is 158 km away from Gondar town, 338 km from Bahir Dar (the capital of Amhara), and 908 km from the country’s capital Addis Ababa.

The town has a total population of 119,054 (63,433 males and 55,617 females) [45]. There are over 120,000 seasonal migrant workers (a majority of males) traveling to Metema for temporary labor and return to their original residence after 3–6 months of stay every year. The town is also noted for its border with Sudan Ethio-Sudan highway. Metema Hospital provides services for over 420,000 catchment population in West Gondar, Northwest Ethiopia, per year. Metema Hospital HIV/AIDS care and treatment clinic was established in 2005. It provides care, treatment, and monitoring for HIV-positive patients as per the national guideline.

### Study population, sample size, and sampling procedure

All adult HIV-positive individuals who were on ART at Metema Hospital and who enrolled from January 1, 2013, to December 30, 2018, were included. However, transferred in-patients with incomplete baseline data and unknown initiation date of treatment and status of patients were excluded. The sample size was estimated using Log-Rank and Cox proportional hazard model in STATA version 14. The calculation was done based on the assumption that type I error of 5%, power of 80% and the exposure is body mass index (BMI) the most significant variable [21]. Survival probability for patients with body mass index greater than 18.5 kg/m^2^ = 0.93. Based on this, the calculated sample size by using STATA 14 software is 542**.** Regarding the sampling technique, a record of study participants have been filtered first from the ART database according to their entry time to follow up, next patients have been filtered using age and eligibility criteria, then we give a unique number for the remaining records and select each record for our study using systematic random sampling.

### Data collection technique and quality control issues

The outcome variable was the time event (death) of patients on ART. The independent variables considered in the study were socio-demographic characteristics such as age, sex, level of education, occupation, marital status, clinical factors like WHO clinical stage, CD4 count, functional status, TB/HIV co-infection, opportunistic infection other than TB, drug adherence, viral load, hemoglobin level, body mass index (BMI) and substance use which is a behavioral factor.

#### Drug adherence

In this study was based on records as Good (equal to or greater than 95% or ≤ 3 doses missed per month), Fair (85–94% or 4–8 doses missed per month) or Poor (less than 85% or ≥ 9 doses missed per month), health workers used reports by the patient or by directly counting the pills to classify adherence. Those patients who adhere greater than or equal to 95% was considered as good adherence; 85–94% fair adherence and Less than 85% was considered as poor adherence [28]. Patient more than three months late for a scheduled visit and who did not return later during follow-up was considered as lost to follow up [29].

The hemoglobin level > 10 gm/dl was categorized as normal hemoglobin and hemoglobin ≤ 10 gm/dl was categorized as low hemoglobin level [30]. In this study, viral load > 1000 copies/ml was recorded as high viral load and viral load ≤ 1000 copies/ml was recorded as low viral load [31]. Substance use was recorded as among those three factors such as alcohol, cigarette smoking, and khat chewing, with a minimum of one record documented in patients' charts or register present.

To assure the data quality, high emphasis was given in designing a data collection instrument. The training was given for data collectors and supervisors to have a common understanding of the purpose of research and the techniques of data collection. A standard checklist was used to record information extracted from electronic and patient cards. Before the actual data collection pretest was done on 25 patient charts, those charts were not included in the study. A standard checklist was used for recording information from the database and patient cards. This form was developed using the standardized ART entry and follow-up form employed by the ART clinic. Three experienced ART Nurses who were trained on comprehensive HIV care were involved in data collection. Throughout the data collection process, completeness, consistency, and accuracy were checked by supervisors and the principal investigator daily.

### Data management and analysis

The data was entered in Epi-data 3.1 and exported to STATA 14 for analysis. Descriptive statistics including proportions, median, tables, and charts was done to describe the characteristics of study participants. Nonparametric estimations of Kaplan–Meier was done graphically and Log-rank test was used to compare the survival probabilities of categorical variables. After fitting the survival model, the proportional hazard assumption was checked using a graphical and global test (goodness test). The model goodness of fit was checked by using the Cox-Snell residual. Cox proportional and parametric models were compared using Akaike Information Criteria (AIC) or Bayesian Information Criteria (BIC). Weibull model was selected since they have low value of AIC and had a good fitness of the Cox Snell residual. Those covariates with a p-value < 0.2 in the bi-variable analysis were again fitted to the multivariable Weibull regression analysis. Both crud and adjusted hazard ratio with the corresponding 95% Confidence Interval (CI) was calculated to show the strength of association. In multivariable analysis, covariates with a p-value of < 0.05 were considered statistically significant.

## Results

### Baseline Scio-demographic, Clinical, and treatment-related characteristics

A total of 542 HIV infected patients on ART were included in this study. Among those, more than half of the 285 (52.6%) study participants were females. The median age of study participants was 32 (IQR = 26–39) years. Nearly two-thirds of the study participants were not educated. Almost half of the 278 (51.3%) study participants disclosed their HIV status and nearly half of the 274 (50.5%) study subjects are co-infected with tuberculosis (TB) (Table 1). Among the study subjects, 179 (33.0%) had a baseline CD4 count < 200 cells/mm^3^, and the baseline WHO staging 2470 (45.6%) of patients were at stage I followed by stage III and stage IV 115 (21.2%). Most of the 413 (76.2%) of the study participants had working functional status and three-fourths of the study subjects had good treatment adherence (Table 1).

**Table 1:** Baseline socio-demographic and clinical characteristics of adults HIV positive patients on ART at Metema Hospital from January 2013 to December 2018

Baseline variable	Survival status					
	Censored		Dead		Total	
	N	%	N	%	N	%
Sex						
Male	204	44.5	53	63.1	257	47.4
Female	254	55.5	31	36.9	285	52.6
Age (years)						
15–24	60	13.1	7	8.3	67	12.4
25–34	211	46.1	28	33.3	239	44.1
35–44	118	25.8	24	28.6	142	26.2
≥ 45	69	15.0	25	29.8	94	17.3
Education						
Not educated	267	58.3	60	71.4	327	60.3
Primary	118	25.8	15	17.9	133	24.5
Secondary & above	73	15.9	9	10.7	82	15.2
Marital status						
Single	102	22.3	22	26.2	124	22.5
Married	190	41.5	28	33.3	218	40.3
Divorced	117	25.6	24	28.6	141	26.1
Widowed	19	4.1	2	2.4	21	4.0
Separated	30	6.5	8	9.5	38	7.1
Substance use						
Yes	38	9.8	39	46.4	77	16.4
No	348	90.2	45	53.6	393	83.6
Occupation						
Daily laborer	170	37.1	45	53.6	215	39.7
Farmer	111	24.2	20	23.8	131	24.2
Housewife	95	20.7	15	17.9	110	20.3
Merchant	61	13.3	2	2.4	63	11.6
Others	21	4.7	2	2.3	23	4.2
Hemoglobin level (g/dl)						
≤ 10	74	16.2	49	58.3	123	22.7
> 10	384	83.8	35	41.7	419	77.3
Viral load (copy/ml)						
≤ 1000	395	88.6	22	37.9	417	82.7
> 1000	51	11.4	36	62.1	87	17.3
CD4 (cells/m^3^)						
≤ 200	116	25.3	63	75.0	179	33.0
201–350	109	23.8	7	8.3	116	21.4
≥ 351	233	50.9	14	16.7	247	45.6
TB-co-infection						
Yes	206	45.0	68	81.0	274	50.5
No	252	55.0	16	19.0	268	49.5
BMI (kg/m^2^)						
≥ 18.5	284	62.0	36	42.9	320	59.0
15.5–18.4	119	26.0	28	33.3	147	27.2
≤ 15.4	55	12.0	20	23.8	75	13.8
WHO stage						
Stage I	234	51.1	13	15.4	247	45.6
Stage II	51	11.1	14	16.7	65	12.0
Stage III	94	20.5	21	25.0	115	21.2
Stage IV	79	17.3	36	42.9	115	21.2
Functional status						
Working	374	81.7	39	46.4	413	76.2
Ambulatory	40	8.7	18	21.4	58	10.7
Bedridden	44	9.6	27	32.2	71	13.1
Adherence						
Good	340	74.2	67	79.8	407	75.0
Fair	64	14.0	3	3.5	67	12.4
Poor	54	11.8	14	16.7	68	12.6
HIV disclosure						
Yes	239	52.2	39	46.4	278	51.3
No	219	47.8	45	53.6	264	48.7
Other OIs						
Yes	40	8.7	27	32.1	67	12.4
No	418	91.3	57	67.9	475	87.6

### Incidence of mortality

**Fig. 1:**
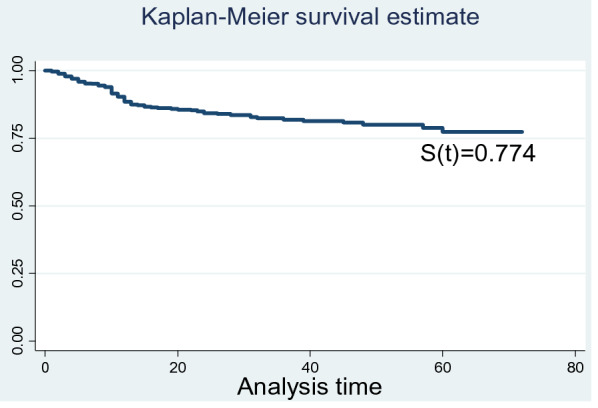
Cumulative survival probability of the patients on ART at Metema Hospital from January 1, 2013 to December 30, 2018

The study subjects followed a total of 72 months after initiation of treatment with a total observation time of 1245 person years. During those total follow up period, the overall incidence rate of mortality was 6.7 (95% CI (5.4–8.4)) per 100-person year of observation. Over the 72 months follow-up period; 4.9 per 100 adult-months (95% CI 3.7–6.3) died during the first 12 months and 0.7 per 100 adult-months (95% CI 0.4–1.1) died during the second year of follow-up. The cumulative survival probability of patients on ART was 77.44% at the end of the study period (Fig. 1).

### Comparison of survival experience by different factors

**Fig. 2:**
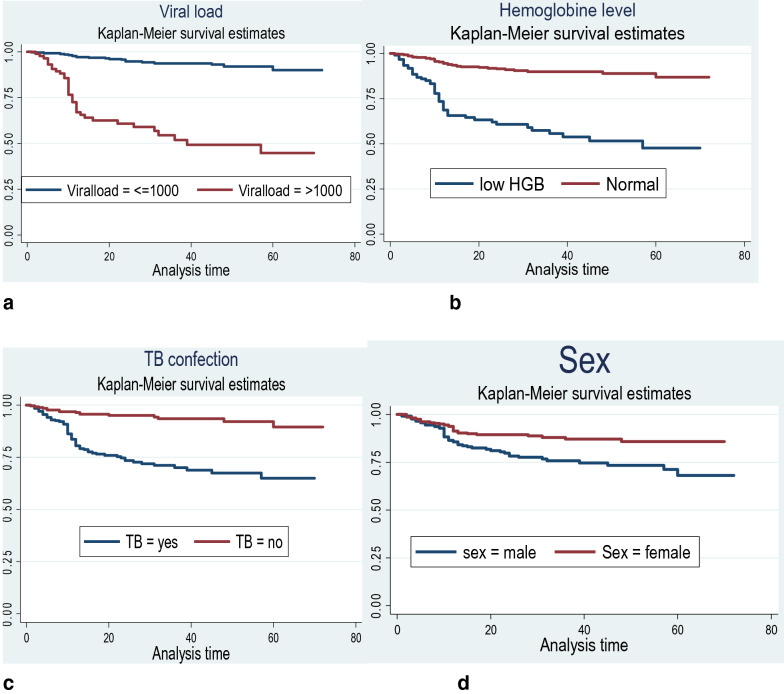
Survival curves for the follow-up of adults on ART according to their Hemoglobin level, viral load, sex & TB-confection at starting of ART

Using the Kaplan–Meier survival curve, the survival experience of the patients was assessed with different categories of predictor variables. Patients with normal hemoglobin had a longer survival experience than those adults with low hemoglobin (Fig. 2) and the difference were statistically significant (Log-Rank, p < 0.001) (Table 2). The observed difference of longer survival of patients with a viral load below threshold compared with those adults above the threshold was found to be statistically significant (Log-Rank, p < 0.001) (Table 2). Patients with TB-co-infection at the era of ART were shorter survival experience than those adults with no TB co-infection (Fig. 2) and the difference were statistically significant (Log-Rank p < 0.001) (Table 2).

**Table 2:** Survival experience comparison with time and Log-Rank value among HIV/AIDS patients on ART at Metema Hospital, Jan 1, 2013 to Dec 30, 2018

Variables	Meantime(by month)	95% CI		Log-Rank p-value
		Lower	Upper	
Sex				
Male	26	24	29	0.0013
Female	30	27	32	
Age (years)				
15–24	25	21	29	0.0075
25–34	29	27	31	
35–44	27	24	31	
≥ 45	29	24	33	
Education level				
No educated	27	25	29	0.0446
Primary	29	26	32	
Secondary & above	32	27	36	
Marital status				
Single	25	22	28	0.3807
Married	31	28	34	
Divorced	24	21	27	
Widowed	27	19	35	
Separated	36	28	45	
Substance use				
Yes	21	16	25	< 0.001
No	29	27	31	
Occupation				
Daily laborer	25	22	27	0.0032
Farmer	27	24	31	
Housewife	30	26	33	
Merchant	30	26	32	
Others	35	29	40	
Hemoglobin (g/dl)				
≤ 10	24	20	27	< 0.001
> 10	29	27	31	
Viral load (copy/ml)				
≤ 1000	30	29	32	< 0.001
> 1000	24	11	28	
CD4 count				
≤ 200 (cells/m^3^)	25	22	27	< 0.001
201–350 (cells/m^3^)	34	30	38	
≥ 351	28	25	30	
TB				
Yes	24	22	27	< 0.001
No	32	29	34	
BMI (kg/m^2^)				
≥ 18.5	29	27	31	< 0.002
15.5–18.4	26	23	29	
≤ 15.4	28	24	33	
WHO clinical stage				
Stage I	29	26	31	< 0.001
Stage II	30	25	35	
Stage III	28	24	31	
Stage IV	26	22	29	
Functional status				
Working	29	27	31	< 0.001
Ambulatory	26	21	32	
Bedridden	26	21	31	
Adherence				
Good	28	26	30	0.026
Fair	29	24	33	
Poor	26	21	30	
Disclosure				
Yes	31	28	33	0.1506
No	25	23	28	
Other OI				
Yes	28	23	33	< 0.001
No	28	26	30	

### Assessing proportional hazard assumption and model selection criteria

**Fig. 3:**
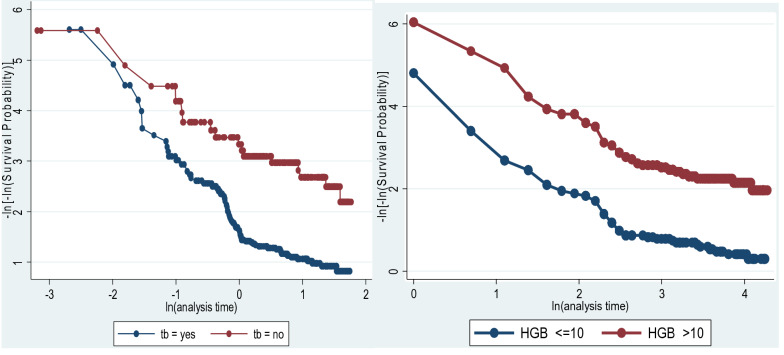
Plot of ln (-ln (survival probability)) versus ln (survival time) for assessing proportional hazard of hemoglobin level and TB co-infection of patients at Metema Hospital, Ethiopia, from January 1, 2013 to December 30, 2018.

Graphical assessment of the proportional hazard assumption was done by comparing the estimated ln(–ln) survivor curves versus ln (survival time) over different categories of variables. The graph of the two categories of tuberculosis and hemoglobin level hazards does not cross each other, which means that the proportional hazard assumption was satisfied (Fig. 3). An additional global test of proportional-hazard assumption based on the Schoenfeld residuals was done and all independent variables satisfy the proportional hazard assumption (Chi square = 11.76, p-value = 0.6971) (Table 3). The model with the smallest value of AIC and BIC was selected. Due to these criteria, the Weibull model was selected and found to be appropriate for this study (Additional file 1).

**Table 3:** Test result of proportional-hazard assumption for predictor variables of mortality among ART- experienced patients at Metema Hospital, from January 2013 to December 2018

Variables	rho	Chi^2^	Df	Prob > Chi^2^
Age	− 0.06467	0.42	1	0.5161
Sex	− 0.10241	0.67	1	0.4121
Education	− 0.05530	0.20	1	0.6525
HGB	− 0.00172	0.00	1	0.9884
Viral load	0.06474	0.28	1	0.5986
CD4	0.06057	0.29	1	0.5897
TB	0.10124	0.59	1	0.4439
BMI	0.00815	0.00	1	0.9446
WHO stage	0.07673	0.34	1	0.5609
Functional status	− 0.10651	0.65	1	0.4185
Adherence	− 0.11155	0.81	1	0.3691
Disclosure	0.21609	3.19	1	0.0740
Other OI	0.07467	0.47	1	0.4943
Occupation	− 0.19293	2.52	1	0.1122
Marital status	− 0.06543	0.25	1	0.6162
Global test		11.76	15	0.6971

**Fig. 4:**
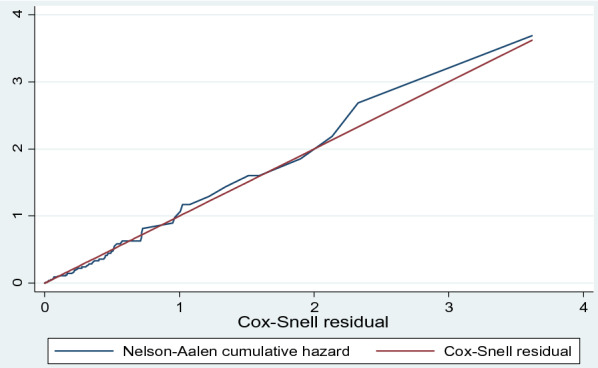
Cox Snell graph for checking model adequacy of Weibull

The Cox-Snell residuals (together with their Nelson-Aalen cumulative hazard function) were done to check the goodness of fit for all models. The figure shows that the plot of the Nelson Aalen cumulative hazard function with Cox-Snell follows the 45° line closely. Hence, the output shows Cox–Snell residuals of the Weibull model were satisfied the overall model fitness compared with other models. Among all models, the Weibull model was appropriately fitted data compared with others (Fig. 4).

### Predictors of mortality among adult patients on ART

After fitting a bi-variable hazard regression model, most predictor variables, i.e., age, sex educational level, occupation, hemoglobin level, viral load, TB co-infection, WHO stage, functional status, adherence, disclosure status OI other than TB and BMI were found to have p-value < 0.2 and entered into multivariable analysis and variables such as sex, hemoglobin level, viral load, WHO clinical stage, and TB co-infection were found to be significant predictors of mortality at 5% level of significance.

In our study, the effect of other variables was constant, the hazard of death among males was 2.4 (HR = 2.4; 95% CI (1.24, 4.62)) times higher than females. Patients with low hemoglobin patients were 4.14 (HR = 4.14; 95% CI (2.18–7.86)) times higher risk of death than those with normal hemoglobin. The hazard of death among patients who had a high viral load was 6.7 ((HR = 6.70; 95% CI (3.40–13.22)) times higher than that of low viral load. Patients at baseline on ART advanced CLINICAL stage IV had 5.64 (HR = 5.64, 95% CI (2.53–12.56)) times increased risk of mortality compared to those presenting with WHO clinical Stage I at the initiation of ART (Table 4).

**Table 4:** Hazard ratios from the single variable and multivariable analysis of the association between the possible predictors of mortality

Independent variables	CHR (95% CI)	AHR (95% CI)
Age in years		
15–24	1.00	1.00
25–34	0.99 (0.43–2.27)	0.70 (0.25–1.92)
35–44	1.51 (0.65–3.50)	1.11 (0.37–3.31)
≥ 45	2.29 (0.99–5.31)	2.43 (0.81–7.31)
Sex		
Female	1.00	1.00
Male	2.10 (1.35–3.27)	2.40 (1.24–4.62)^***^
Education level		
No education	1.94 (0.96–3.90)	0.68 (0.26–1.79)
Primary	1.10 (0.48–2.52)	0.59 (0.19–1.82)
Secondary & above	1.00	1.00
Hemoglobin		
Low	5.73 (3.71–8.85)	4.14 (2.18–7.86)***
Normal	1.00	1.00
Viral load		
≤ 1000	1.00	1.00
> 1000	9.84 (5.78–16.73)	6.70 (3.40–13.22)***
CD4		
≤ 200	6.90 (3.87–12.31)	1.71 (0.77–3.76)
201–350	0.89 (0.36–2.20)	0.53 (0.16–1.76)
> 351	1.00	1.00
TB coinfection		
Yes	5.24 (3.04–9.05)	3.71 (1.59-8.64)***
No	1.00	1.00
BMI in kg/m^2^		
≥ 18.5	1.00	1.00
15.5–18.4	1.88 (1.15–3.09)	1.07 (0.51–2.22)
≤ 15.4	2.43 (1.41–4.20)	2.45 (1.17–5.10)***
WHO clinical stage		
Stage I	1.00	1.00
Stage II	3.92 (1.84–8.34)	2.58 (0.94–7.07)
Stage III	3.63 (1.82–7.24)	3.31 (1.35–8.10)***
Stage IV	6.52 (3.46–12.30)	5.64 (2.53–12.56)***
Functional status		
Working	1.00	1.00
Ambulatory	3.57 (2.04–6.23)	1.04 (0.48–2.24)
Bedridden	4.40 (2.70–7.18)	0.93 (0.45–1.94)
Adherence		
Good	1.00	1.00
Fair	0.27 (0.08–0.86)	0.38 (0.08–1.70)
Poor	1.37 (0.77–2.43)	0.92 (0.40–2.09)
Disclosure status		
Yes	1.00	1.00
No	1.43 (0.93–2.19)	0.84 (0.45–1.58)
OIs other than TB		
Yes	3.41 (2.16–5.39)	1.19 (0.55–2.55)
No	1.00	1.00
Occupation		
Merchant	1.00	1.00
Daily laborer	8.86 (2.15–36.54)	5.57 (0.71–43.94)
Farmer	5.97 (1.39–25.53)	3.81 (0.45–32.01)
Housewife	4.90 (1.12–21.44)	5.74 (0.68–48.23)
Others^a^	2.62 (0.37–18.56)	0.45 (0.02–9.14)

_HR: Hazard ratio; CI: Confidence interval; OIs: Opportunistic infection; 1: reference; BMI: body mass index_

^***^_Indicates that the variables significantly associated with the outcome at a 95% level of significance (P < 0.005)_

^a^_Indicates that driver, students, and jobless_

## Discussion

This study aimed to assess the incidence and predictors of mortality among adult HIV patients on ART at Metema Hospital, Northwest Ethiopia. Many other studies presented different predictors for mortality, and our study also assessed the magnitude of mortality and corresponding socio-demographic, clinical, and treatment-related factors. In this study, the overall incidence rate of mortality among adult HIV/ADIS patients who are on ART was 6.7 (95% CI (5.4–8.4)) per 100-person year of observation, consistent with other studies conducted in different areas of Ethiopia [20, 27]. This might be due to the implementation of ART services based on the ART guideline. However, the current finding of this study is higher than in other studies of Ethiopia [18, 19, 22, 23, 25, 26]. This difference might be due to our study was conducted in large number of migrant workers and daily laborers since daily laborers pass their time in the whole day in the work place and they do not have enough time to come health facility for medication and follow up. Because of the nature work, they are also more mobile and have no constant workplace that may end up with loss to follow up and poor treatment adherence which promotes death. Also, this group of population had low income (unable to feed themselves) and they believe that taking ART medication on an empty stomach is difficult and dangerous that leads them to seek an alternative options like holy water by stopping ART which facilitates death. However, the current finding is lower than the findings in Cameroon [32] and Uganda [33]. This might be due to a variation in the ART enrollment period with different treatment eligibility criteria and there was a variation in the implementation of new WHO treatment initiation recommendations in Sub-Saharan countries [34].

In this study, the hazards of death among male patients were 2.4 times higher than females in line with other studies [13, 35, 36], systematic review and meta-analysis conducted in low and middle-income country [14] and south Africa [37]. Since females have a better opportunity to practice the early diagnosis of HIV/AIDS, such as at a time of pregnancy, females were screened for HIV tests as a part of the PMTCT program. Besides this, a higher risk of mortality in males might be due to behavioral factors of males like substance use, potentially leading to late HIV diagnosis and poor adherence to ART. Also, another study assessing mortality rate among HIV positive patients in Uganda indicated that male patients 37% more risk of mortality compared to female patients at the starting of ART [13].

In this study, those ART clients with higher viral load had an increased hazard of death in agreement with the study conducted in seven teaching Hospitals of Ethiopia [27]. This could be a lower rate of viral suppression and poor immunological recovery at baseline.

Similarly, patients who had advanced WHO clinical stages (stage III and IV) were a higher hazard of death than those in the WHO clinical stage I consistency with studies conducted in Sub-Saharan Africa [12, 38–41]. This reflects the advanced immunodeficiency increases the risk of mortality.

Patients who had low hemoglobin levels have an increased hazard of death than those who had normal hemoglobin levels. This finding is comparable with the studies conducted in Sub-Saharan Africa [8, 33, 42–44]. This might be the HIV is directly affecting the bone marrow and the high incidence of anemia might increase with the progression of HIV infection, which probably has contributed to the increasing the risk of mortality. Moreover, anemia among HIV patients can lead to impaired physical functioning, psychological distress, poor quality of life, accelerated disease progression, and shorter life expectancy [30].

Patients with TB-co infected with increased the hazard of death compared to clients not developing TB. This finding is consistent with studies conducted in rural Uganda [33] and in Ethiopia [21, 22]. This might be due to host responses to M. tuberculosis enhance HIV replication, accelerating the natural progression of HIV, and further depressing cellular immunity [45]. Decreased gut absorption of anti-tuberculosis drugs leads to impaired treatment outcomes including mortality. Furthermore, TB and HIV is double burden infection which leads to cause of mortality worldwide [46].

In this study, the hazard of death among patients with BMI ≤ 15.4 kg/m^2^ was 2.4 times higher than those with a BMI ≥ 18.5 kg/m^2^. This study is similar to studies conducted in Tanzania [8], Ethiopia [22], Cameroon [32], Uganda [33], and Malawi [38]. Patients with lower BMI are malnourished and unable to cope with the disease and had a high exposure to opportunistic infections that leads to death [47].

## Limitation of study

The potential limitation of this study was because of the retrospective nature of the study which lacks completeness of patient records like substance use, provision of opportunistic infection, prophylaxis status, and hospitalization of patients. We could not ascertain that all recorders of mortality were AIDS-related, so all mortality was considered as HIV/ AIDS-related because of the lack of records on the cause of mortality.

## Conclusion

In this study, the incidence of mortality was high. Patients on ART who have Advanced WHO patients (stage III and IV), viral load > 1000 copy /ml, hemoglobin ≤ 10 mg/dl, TB co-infection, BMI ≤ 15.4 kg/m^2^, and being male sex had a higher risk of mortality. Special attention should be given to male patients and high public interventions needed among HIV patients on ART to reduce the mortality rate. Nutritional support and close monitoring of patients in the early period of ART treatment initiation is very vital to improve patient survival.

## Supplementary Information

**Additional file 1.** AIC and BIC value for model comparison of Cox and parametric model

## Data Availability

The datasets used and/or analyzed during the current study are available from the corresponding author on reasonable request.

## Abbreviations

AIC: Akaike Information Criteria
AIDS: Acquired Immune Deficiency Syndrome
ART: Antiretroviral Therapy
BIC: Bayesian Information Criteria
MI: Body mass index
HAART: Highly active antiretroviral therapy
Hgb: Hemoglobin
OI: Opportunistic infection
PMTCT: Prevention of Mother to Child Transmission
PYO: Person Year Observation
UNAIDS: United Nations Joint Program on HIV/AIDS
WHO: World Health Organization
