# Supplementary material for: Predictors of mortality rate among adult HIV-positive patients on antiretroviral therapy in Metema Hospital, Northwest Ethiopia: a retrospective follow-up study
Source: AIDS Res Ther. 2021 May 5;18:27. doi: 10.1186/s12981-021-00353-z (PMC8097881; doi:10.1186/s12981-021-00353-z)
Supplement: Supplementary file 1 — Additional file 1. AIC and BIC value for model comparison of Cox and parametric model [file 12981_2021_353_MOESM1_ESM.docx]

| **Model** | **Observation** | **ll (null)** | **ll (model)** | **df** | **AIC** | **BIC** |
| --- | --- | --- | --- | --- | --- | --- |
|  |  |  |  |  |  |  |
| Cox | 504 | -337.407 | -252.6206 | 15 | 535.2413 | 598.5799 |
| Exponential | 504 | -236.4307 | -148.4196 | 16 | 328.8392 | 396.4004 |
| Weibull | 504 | -236.0156 | -145.0328 | 17 | 324.0657 | 395.8495 |
| Gompertze | 504 | -233.189 | -148.1046 | 17 | 330.2091 | 401.9929 |
| Lognormal | 504 | -232.4004 | -150.4255 | 17 | 334.8511 | 406.6349 |
| Log logistic | 504 | -235.2821 | -147.7239 | 17 | 329.4477 | 401.2315 |
| Generalized gamma | 504 | -256.233 | -229.4639 | 18 | 464.9278 | 477.5955 |
|  |  |  |  |  |  |  |
